# Supplementary material for: Taxonomic analysis of metagenomic data with kASA
Source: Nucleic Acids Res. 2021 Mar 30;49(12):e68. doi: 10.1093/nar/gkab200 (PMC8266618; doi:10.1093/nar/gkab200)
Supplement: gkab200_Supplemental_Files [file gkab200_supplemental_files.zip › Suppl_D.zip!/OPAL_comparison_new/gold_standard/superkingdom.pdf]

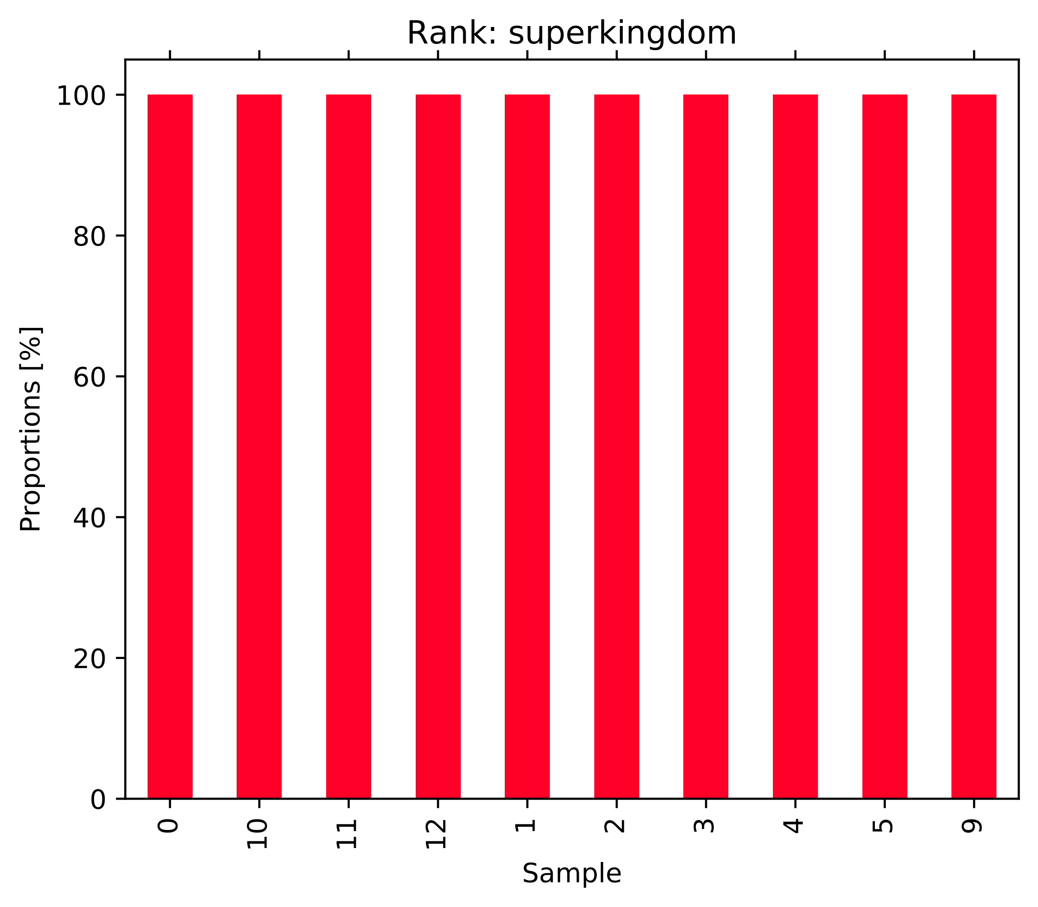
Rank: superkingdom
Proportions [%]
100
80
60
40
20
0
0
10
11
12
1
2
3
4
5
9
Sample
